# Funding Opportunity in Functional Target Validation for ADRD

**DOI:** 10.1002/alz.095380

**Published:** 2025-01-09

**Authors:** Pascal Laeng, Charles Cywin

**Affiliations:** ^1^ NIH‐NINDS, North bethesda, MD USA; ^2^ NINDS, Bethesda, MD USA

## Abstract

**Background:**

Although the success rate of novel treatments for CNS diseases is low, this has not limited the enthusiasm of academic and industry neuroscientists to undertake novel drug discovery approaches for the treatment of Alzheimer’s Disease‐Related Dementias (ADRD) due to the significant unmet need. A first major obstacle in the development of promising treatments for ADRD and other CNS disorders is the need for well‐validated targets linked to the disease process. Partial and incomplete knowledge about the biology of the selected molecular target(s) often limit their chance to translate into successful drug discovery projects. Translating these discoveries to the clinic will depend on the quality of the target validation process and the confidence in a causative link between the modulation of a target(s) and its functional physiological consequence(s) associated with treating or preventing a disease state. Recent technological innovations provide new opportunities in developing new converging approaches to validate the molecular and physiological functions of novel target candidates and their causality in disease progression.

**Method:**

NINDS, in partnership with NIA, is supporting new funding opportunity in functional target validation for ADRD to increase confidence in the efficacy and safety of novel emerging target(s) to trigger the development of customized therapeutic strategies. This will be implemented by a NOFO to fund the development of customized technologies, models, and protocols to modulate the expression (or activity) of ADRD target candidate(s) and monitor their functional biological consequences in *in vitro* and *in vivo* disease models. To inform future therapeutic efforts, positive as well as the negative functional impacts of the target modulation need to be tested in different modalities and across collaborating laboratories.

**Result:**

NINDS invites applications to propose the comprehensive functional validation of newly identified therapeutic target candidates for ADRD. This poster will outline in more detail, the eligibility requirements for the target candidate, potential druggability and knowledge gaps as well as the budget and the research objectives and scope.

**Conclusion:**

This funding opportunity will provide the translational research community with increased confidence in the efficacy and safety of the novel emerging target(s).

